# Combining machine learning with structure-based protein design to predict and engineer post-translational modifications of proteins

**DOI:** 10.1371/journal.pcbi.1011939

**Published:** 2024-03-14

**Authors:** Moritz Ertelt, Vikram Khipple Mulligan, Jack B. Maguire, Sergey Lyskov, Rocco Moretti, Torben Schiffner, Jens Meiler, Clara T. Schoeder

**Affiliations:** 1 Institute for Drug Discovery, Leipzig University Medical Faculty, Leipzig, Germany; 2 Center for Scalable Data Analytics and Artificial Intelligence ScaDS.AI, Dresden/Leipzig, Germany; 3 Center for Computational Biology, Flatiron Institute, New York, New York, United States of America; 4 Program in Bioinformatics and Computational Biology, University of North Carolina at Chapel Hill, Chapel Hill, North Carolina, United States of America; 5 Department of Chemical and Biomolecular Engineering, Johns Hopkins University, Baltimore, Maryland, United States of America; 6 Department of Chemistry, Vanderbilt University, Nashville, Tennessee, United States of America; 7 Center for Structural Biology, Vanderbilt University, Nashville, Tennessee, United States of America; University of Kansas, UNITED STATES

## Abstract

Post-translational modifications (PTMs) of proteins play a vital role in their function and stability. These modifications influence protein folding, signaling, protein-protein interactions, enzyme activity, binding affinity, aggregation, degradation, and much more. To date, over 400 types of PTMs have been described, representing chemical diversity well beyond the genetically encoded amino acids. Such modifications pose a challenge to the successful design of proteins, but also represent a major opportunity to diversify the protein engineering toolbox. To this end, we first trained artificial neural networks (ANNs) to predict eighteen of the most abundant PTMs, including protein glycosylation, phosphorylation, methylation, and deamidation. In a second step, these models were implemented inside the computational protein modeling suite Rosetta, which allows flexible combination with existing protocols to model the modified sites and understand their impact on protein stability as well as function. Lastly, we developed a new design protocol that either maximizes or minimizes the predicted probability of a particular site being modified. We find that this combination of ANN prediction and structure-based design can enable the modification of existing, as well as the introduction of novel, PTMs. The potential applications of our work include, but are not limited to, glycan masking of epitopes, strengthening protein-protein interactions through phosphorylation, as well as protecting proteins from deamidation liabilities. These applications are especially important for the design of new protein therapeutics where PTMs can drastically change the therapeutic properties of a protein. Our work adds novel tools to Rosetta’s protein engineering toolbox that allow for the rational design of PTMs.

## Author summary

Machine learning is changing the world of protein design, from structure prediction methods like AlphaFold to fixed-backbone design methods like ProteinMPNN. machine learning methods have made much progress in various aspects of protein computational biology, both complementing and, in some cases, surpassing traditional macromolecular modeling methods such as those combined in libraries like the Rosetta software suite. However, a lack of compatibility and flexibility can hinder interoperability with existing methods, preventing the full potential of these new solutions from being realized. Here, we first present a new machine learning tool for predicting post-translational modifications (PTMs), which play an important role in the stability and function of proteins, and then highlight how the implementation of this tool in the existing Rosetta toolbox can facilitate new applications. To this end, we combine PTM prediction with protein design, maximizing or minimizing the predicted probability of a post-translational modification occurring at a specific site. As one example, we predict the *N*-linked glycosylation of influenza hemagglutinin, which has applications in both understanding the evolution of viral strains over time, and engineering additional glycosylation sites to mask unwanted epitopes of vaccine candidates.

This is a *PLOS Computational Biology* Methods paper.

## Introduction

PTMs play an important role in modulating both protein stability and many aspects of protein function. PTMs can be divided into reversible and irreversible modifications, with some modifications, like *N*-linked glycosylation, even occurring before protein folding. The diversity of possible PTMs highlights the complex chemical composition of proteins, which is not limited to the standard 20 letter amino acid code. Understanding the impact of modifications is especially vital in the field of protein therapeutics, where PTMs can range from being essential for desired therapeutic function, to completely blocking therapeutic function through unforeseen changes in stability and function over time [1].

Glycosylation describes the enzymatic attachment of an oligosaccharide to a protein residue. This generally occurs in the endoplasmic reticulum (ER) and Golgi apparatus for proteins bound for secretion or for cell surface expression, though rare cytoplasmic and nuclear glycoproteins are known [2,3]. Glycosylation is further classified into *N*-and *O*-glycosylation, where the carbohydrate linkage occurs either at the side-chain amide nitrogen of an asparagine residue (*N*-glycosylation), or the hydroxyl oxygen atom of a serine, threonine, or (very rarely) tyrosine residue (*O*-glycosylation). Additionally, *N*-glycosylation occurs in the unfolded state while *O*-glycosylation occurs after the protein is already folded. Both *N*-and *O*-glycosylation tend to increase thermostability and solubility [4], and both can modulate interactions with other proteins [2]. For *N*-glycosylation, there exists a well-known sequence motif: NxT/S, where x is any amino acid except proline [5]. While this sequence motif is helpful in identifying potential sites, the existence of a sequon is not sufficient to guarantee glycosylation. Additionally, multiple improved sequons have been discovered through trial and error, highlighting the complexity beyond the NxT/S motif [6,7]. For *O*-glycosylation a clear sequence motif is not known; however, *O*-glycosylation sites tend to cluster in proline/serine rich flexible regions of proteins [8]. Glycosylation of protein therapeutics can impact their folding, solubility, thermal stability, chemical stability, and aggregation propensity [9,10,11]. The list of protein drugs affected by glycosylation is long and includes chymotrypsin [12,13], insulin [14], lenograstim [15,16,17], antithrombin [18], agalsidase alfa/beta [19,20,21], and various antibodies [11] (for a detailed review refer to [10]). For this reason, when engineering protein therapeutics, it is essential to be able to predict glycosylation, and extremely useful to be able to rationally design for or against it. This “glycoengineering” can be particularly useful in vaccine development: off-target epitopes, for instance in engineered epitope-presentation scaffolds, can be “masked” by suitable introduction of glycosylation sites [22,23]. Glycans are commonly used by viruses to hide antigenic protein surfaces, however, this mechanism can also be used to prevent unwanted immune reactions in vaccines and direct an immune response to a desired site. For influenza hemagglutinin, for example, the creation of a “hyper-glycosylated” variant through seven additional glycosylation sites lead to better protection against morbidity and mortality in mice upon virus challenge by directing the immune response to a neutralizing epitope left unglycosylated [24].

Deamidation, the spontaneous reaction of asparagine to isoaspartate, is one of the most commonly occurring PTMs known. The resulting modification leads to structural changes through the insertion of a negative charge and through significant alteration of the protein backbone (effectively, replacing an α-*L*-amino acid with a β^3^-amino acid with the chiral center reversed), affecting both protein stability and function. *In vivo*, deamidation is thought to play the role of a molecular “clock”, marking proteins for degradation through increased susceptibility to proteolysis [25,26,27]. The rate of deamidation is not only influenced by pH and temperature but also by its local environment, including the neighboring residues, secondary structure, and solvent accessibility [28,29]. Therefore, the deamidation half-life of a protein can be as long as several months or as short as hours, at which point it can begin to affect the pharmacokinetics of therapeutic proteins. A commonly described deamidation motif is an asparagine in a flexible loop, followed by a glycine residue [28]. The occurrence of a deamidation site, however, cannot be simply derived from sequence alone and thus remains unpredictable without experimental characterization. For therapeutic proteins the rate of deamidation can strongly influence both shelf life and persistence time in the body, through either loss of function or stability, and therefore render them ineffective [30,31]. Therapeutic proteins affected include, but are not limited to, antibodies [32], vaccine antigens [33,34,35], peptides [36], adeno-associated virus (AAV) serotypes used for human gene therapy [37], human hormones [25,38] and enzymes [25]. In case of AAV vectors, multiple deamidation sites were discovered and engineered for enhanced stability against deamidation, leading to higher transduction efficiencies in mice, as well as different T cell activation profiles [39]. For antibodies, deamidation potentially leads not only to aggregation but also to drastic decreases in antigen binding affinity [32]. Deamidation sites are commonly discovered late in the development process and then corrected by trial-and-error mutation studies, leading to unnecessary costs and liabilities. Although nowadays many companies use computational liability screening methods, most of them are purely sequence-based.

More recently, several studies have used rational design to create proteins responsive to changes in either phosphorylation or glutathionylation with potential applications in building biomaterials or controlling cellular behavior. Scheuermann et al. [40] and Gao et al. [41] designed minimal domains derived from EF-Hand calcium-binding domains that only bind terbium upon glutathionylation or phosphorylation of a key residue, therefore regulating the function of a protein through its modification status. Similarly, Winter et al. [42] and Thompson et al. [43] designed proteins with their multimerization status being defined by whether a particular residue located at the interface is phosphorylated or not. Woodall et al. [44] combine the two approaches, creating tyrosine and serine kinase-driven protein switches where protein association is controlled by kinase activity, leading to the reconstitution of green fluorescent protein fluorescence or the inhibition of the protease calpain. These seminal studies highlight the potential benefit of PTM-aware protein engineering.

As the occurrence and rate of PTMs is dependent on multiple factors, prediction needs to take many features into account. Previously, multiple studies used machine learning methods to predict PTMs, generally focusing on a single prominent modification. Often, sequence is the only readily available information, and therefore used as the main feature in combination with *in silico* predicted structural features like solvent accessibility or secondary structure. In the case of protein deamidation, for example, a recent study used both sequence and selected structural features, including neighboring residues, solvent accessible surface area (SASA), dihedral angles and half-life times derived from a mass spectrometry poly-peptide study [27]. In the case of *N*-linked glycosylation, multiple studies [45,46,47,48] trained neural networks on the sequence context of glycosylation sites, often using the full-length protein sequence, or leveraging homology-based features. In these cases, it is not entirely clear whether the model learned general sequon preferences or simply protein homology, especially in the case where proteins with cellular localization in the nucleus or no glycosylation sequon were used as negative examples. However, the usefulness of a predictive model is not measured alone by its accuracy, but whether the choice of data reflects the downstream task the model is intended to be used for. The approaches do not only differ by the features or neural network architectures used, but crucially by their choice and filtering of data. These filtering steps are especially important to avoid overestimating the performance of a model, because of, for example, missed homology or false negatives. While these models are potentially useful for predicting glycosylation in natural proteins, they are of limited use in the case of (re-)engineering proteins. With the recent revolution in protein structure prediction [49,50], however, structural features are more readily available to complement sequence information. The engineering of modification sites would offer both the reduction of liabilities from unwanted PTMs, as well as the introduction of desirable PTMs in order to improve stability or alter functionality of therapeutics. The protein modeling suite Rosetta [51] has proven successful in tasks such as designing proteins for thermodynamic stability [52] and functionality [53]. By implementing accurate prediction of PTMs using machine learning in Rosetta, we can combine this new tool with Rosetta’s existing structure-based protein design toolbox to either screen pools of natural, reengineered, or *de novo* designed proteins for the presence or absence of a PTM, or to impose the presence or absence of a PTM as a requirement during the design process. Moreover, by bringing this into the context of existing protein design protocols, we can combine PTM restrictions or requirements with other design objectives for which well-validated optimization protocols already exist, permitting multi-objective optimization. The integration into the existing Rosetta ecosystem also permits the use of these tools for analytical purposes, to model different modifications in the contexts in which they are likely to occur in order to aid understanding of their impact on protein function and stability. For example, the already present glycosylation modeling tools [54,55,56] allow us to further test the plausibility of a predicted glycosylation site, as well as make predictions about its impact on, for instance, a modelled protein-protein interaction. To our knowledge, no protocol for engineering PTMs which combines machine learning with structure-based design has been implemented yet. We argue that this combination of predictive machine learning methods with structure-based design has great potential for a variety of protein engineering applications [57].

In this study, we implemented both, a metric that scans a given protein structure for predicted PTM sites, as well as a protocol using protein design to either increase or decrease the predicted probability of a modification to occur. Compared to earlier work, we leverage recent improvements in the field of natural language processing, as well as similarities between modifications, to improve the prediction accuracy. Additionally, the models are implemented as a SimpleMetric [54] (in Rosetta, a module for measuring a property of a structure), allowing seamless integration with other RosettaScripts objects. Internally, the implemented SimpleMetric, called the PTMPredictionMetric, accesses the Tensorflow model through Tensorflow’s C API [58]. To ensure robustness, avoid repeated load and initialization of the Tensorflow model, and minimize developer error, we also built a framework, called the RosettaTensorflowManager, for structured C++-style interaction with machine learning models. By implementing these methods in Rosetta, we benefit from existing infrastructure for unit, integration, and scientific testing [59], ensuring that the methods remain functional and that results produced with them remain reproducible. In comparison, Python library compatibilities can be notoriously hard to organize and maintain, hindering reproducibility. A recent study on computational biology webservers found only 31% of them to be consistently working [60].

As a demonstration of our methods, to modify the predicted probability of a modification to occur, we design proteins using a Monte Carlo protocol optimizing the Rosetta score as well as the predicted modification probability. This combination allows us to find a tradeoff between thermodynamic stability and predicted PTM rate. Additionally, given a functionally relevant structure, like an antibody-antigen complex, we can further ensure that the mutation is not disrupting the functionality of a given protein.

## Results

### Collection of sequence and structural data for experimental verified modification sites

**Fig 1:**
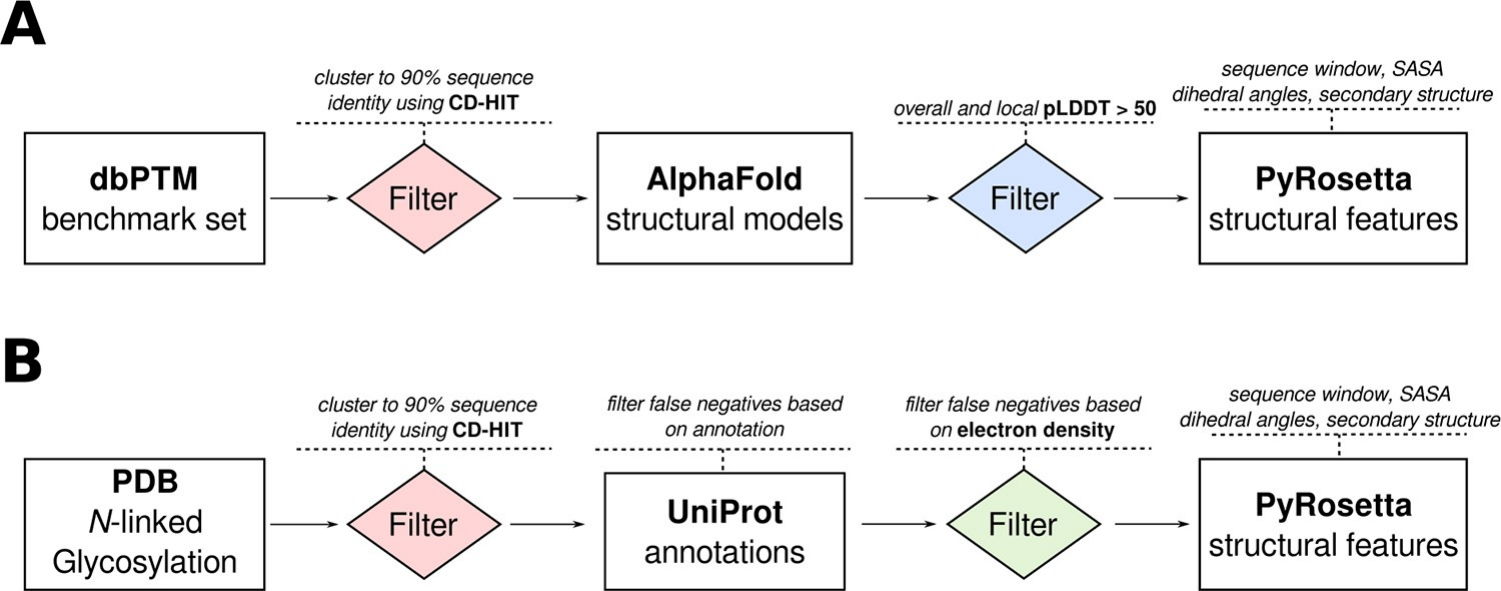
Collection of data and feature calculation. **A)** For all modifications except *N*-linked glycosylation and deamidation, data were collected from the dbPTM and sequence windows of ten residues before/after the modified site were filtered with CD-HIT to 90% sequence identity. Predicted structural models were downloaded from the AlphaFold2 database and filtered by overall and local pLDDT over 50. PyRosetta was used to calculate dihedral angles, secondary structure, and solvent-accessible-surface-area (SASA). **B)** For *N*-linked glycosylation, structures of eukaryotic proteins produced in a eukaryotic expression system with at least one glycan were collected from the Protein Data Bank (PDB) and sequence windows of ten residues before/after the modified site were filtered with CD-HIT to 90% sequence identity. To avoid false negatives, glycosylation sites were compared to UniProt annotations of experimentally verified glycosylation sites and further manually screened for spurious electron density (potentially representing glycan occupancy) or endoglycosidase treatment, removing any such cases from the dataset. PyRosetta was used to calculate the same set of features as for the other modifications.

In order to train a machine learning model to predict PTMs, we first collected experimentally verified modification sites from the dbPTM [61,62], which provides a non-homologous benchmark dataset with positive and negative sites. In addition to the sequence information, we collected predicted structures for each entry from the AlphaFold2 database [49,50] and filtered them by local as well as overall pLDDT. The resulting structures were used to calculate the SASA, dihedral angles and secondary structure of the modified site and its neighbors using PyRosetta [63] (**Fig 1**). For most PTMs, an exact sequence motif is not known and therefore the negative examples are not limited to a particular motif. In the case of *N*-linked glycosylation, however, as the NxT/S sequon (where X is any amino acid except proline) is well-described, we decided to focus on predicting whether each asparagine occurring in a sequon is modified, rather than making predictions for all asparagines. To achieve this, we collected structures of eukaryotic proteins produced in eukaryotic expression systems from the protein data bank (PDB) [64] that have at least one sequon with a resolved glycan and searched them for additional sequons that were not glycosylated. To avoid false negatives, we manually screened electron densities to avoid un-assigned glycosylation sites, filtered proteins treated with an endoglycosyidase (e.g. PNGase F), as well as cross-checked against the UniProt [65,66] database. This selection resulted in 2115 positive and 355 negative samples for *N*-linked glycosylation (Table C in S1 Text). Lastly, as there was no available benchmark dataset for deamidation, we used the published data from Delmar *et al*. [67] to reproduce a deamidation classifier. Since the full sequences of their training data were not publicized, we could not predict their structure with AlphaFold2 and were instead restricted to published data. The number of available data differs drastically for PTMs, for example, phosphorylation has 61340 datapoints while crotonylation has only 145 in total (Table C in S1 Text). The median over all PTMs was 2327 positive and 3690 negative examples. For all datasets, a sequence window around the potentially modified site, instead of the full sequence, was used to prevent signals such as homology to protein families or cellular localization, as we deemed a model fitted to these signals not practically useful in engineering new modifications.

### Prediction of PTMs using machine learning

**Fig 2:**
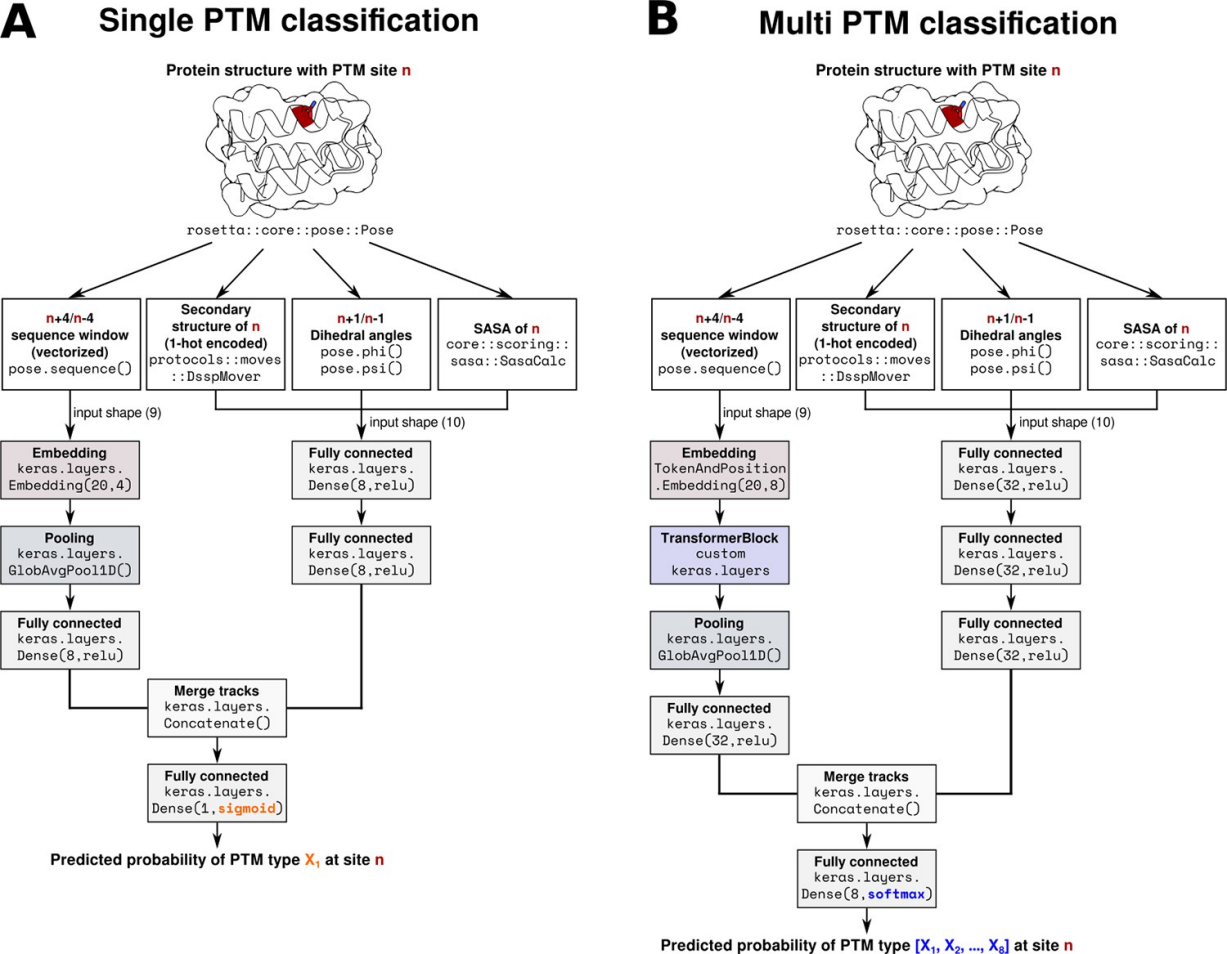
Neural network architecture for predicting post-translational modifications (PTMs). Starting from a Rosetta pose object representing a protein structure and its attributes, sequence and structural features are calculated by already implemented methods in Rosetta and then input into an artificial neural network (ANN) built using the Keras functional API. **A)** Single PTM classification using an embedded sequence window and structural features as input to two-tracks of fully connected layers. Here, one model is trained for each type of PTM. **B)** Multi PTM classification using the same features but with an additional transformer layer in the sequence track and an additional fully connected layer in the structure track of the network. This model combines PTM types with unique amino acids in training and therefore predicts probabilities for multiple PTMs.

To predict the occurrence of PTMs, we first trained a two-track neural network for each modification (**Fig 2**). One track processed sequence features, and the other processed structural features. The resulting Matthews correlation coefficients (MCC) of the test set ranged from 0.76 for proline hydroxylation to 0.10 for *N*-linked glycosylation (**Table 1**). As modifications with little or unbalanced data showed worse performance, we hypothesized that these cases would benefit from training a classifier which combines prediction of multiple PTMs. As some modifications shared the same kind of amino acid, however, we were not able to train one model for all 19 modifications simultaneously, as a negative example of a e.g., lysine succinylation modification is not guaranteed to also not be sumoylated. Therefore, we trained a multi-prediction model for each modification including all other unique amino acid modifications (Table 1), excluding PTMs affecting the same kind of amino acid. For example, to predict crotonylation (Lys) a model was trained to simultaneously predict hydroxylation (Pro), γ-carboxyglutamic-acid modification (Glu), arginine methylation (Arg), glutathionylation (Cys), phosphorylation (Ser/Thr) and *N*-linked glycosylation (Asn) but not any other lysine modification. In the case of crotonylation, this increases the training data from just 145 crotonylation examples (23 positive, 122 negative) to 88103 examples (11452 positive, 76651 negative) of different PTMs. Multiple PTMs showed a clear improvement over the single prediction case, for example the MCC of *N*-linked glycosylation increased from 0.1 to 0.2 and the MCC of crotonylation increased from 0.32 to 0.49. Overall, modifications with few data improved the most from the multi-prediction approach. For deamidation, we use the published data by Delmar et al. [67] which does not include full sequences or structures and was not made available upon request. Therefore, we were limited to the original data in our feature set and could not combine it with other PTMs.

**Table 1:** Different model performances on post-translational modifications.

PTM	AA type	MCC single	MCC multi	AUC single	AUC multi	FP single	FP multi	FN single	FN multi
**Hydroxylation**	**P**	**0.76**	0.75	**0.96**	0.88	**0.08**	0.12	**0.16**	0.12
**γ-carboxyglutamic-acid**	**E**	0.53	**0.67**	0.85	**0.83**	0.27	**0.17**	0.21	**0.17**
**Deamidation**	**N**	**0.54**	NA	**0.85**	NA	**0.09**	NA	**0.31**	NA
**Lys Methylation**	**K**	0.41	**0.41**	0.78	**0.70**	0.27	**0.22**	0.31	**0.37**
**Malonylation**	**K**	**0.37**	0.30	**0.75**	0.65	**0.29**	0.34	**0.33**	0.36
**Arg Methylation**	**R**	0.37	**0.40**	0.79	**0.71**	0.23	**0.16**	0.36	**0.43**
**Crotonylation**	**K**	0.32	**0.49**	0.77	**0.74**	0.48	**0.44**	0.17	**0.08**
**Ubiquitination**	**K**	**0.31**	0.26	**0.72**	0.63	**0.36**	0.37	**0.32**	0.37
**Succinylation**	**K**	**0.31**	0.22	**0.71**	0.61	**0.39**	0.39	**0.30**	0.40
**Glutathionylation**	**C**	**0.30**	0.20	**0.70**	0.60	**0.40**	0.44	**0.30**	0.36
**Sumoylation**	**K**	0.30	**0.30**	0.76	**0.66**	0.32	**0.22**	0.30	**0.45**
***S*-Nitrosylation**	**C**	**0.28**	0.17	**0.70**	0.58	**0.40**	0.34	**0.31**	0.50
**Acetylation**	**K**	**0.22**	0.22	**0.68**	0.62	**0.44**	0.49	**0.31**	0.28
***O*-linked Glycosylation**	**S/T**	0.21	**0.27**	0.75	**0.72**	0.22	**0.20**	0.42	**0.35**
**Phosphorylation**	**S/T**	0.17	**0.24**	0.76	**0.72**	0.28	**0.16**	0.34	**0.39**
**Glutarylation**	**K**	0.12	**0.18**	0.58	**0.60**	0.45	**0.45**	0.42	**0.35**
**Citrullination**	**R**	0.10	**0.12**	0.56	**0.61**	0.13	**0.20**	0.74	**0.58**
***N*-linked Glycosylation**	**N**	0.10	**0.20**	0.57	**0.62**	0.49	**0.50**	0.37	**0.25**

Better performing model in bold; AA, Amino Acid; MCC, Matthew’s correlation coefficient; AUC, Area under the curve of the receiver operating characteristic curve; FP, False Positive rate; FN, False Negative rate

All models were trained using the Tensorflow library [58] which offers a broad array of solutions for implementing models in a production setting. The models were converted into Tensorflow graphs that can be loaded and used for inference through the Tensorflow C API that is wrapped in Rosetta by a special RosettaTensorflowManager. This is described in detail in **S1 Text**.

After training the prediction classifiers and integrating them in the Rosetta suite, we set out to combine the prediction with protein design to influence the predicted probability of a modification occurring at a given site. To demonstrate this, we chose *N*-linked glycosylation and deamidation as examples of either preventing or introducing a particular PTM using protein design.

### Predicting deamidation propensity of Protein A mutations using structure-based design

**Fig 3:**
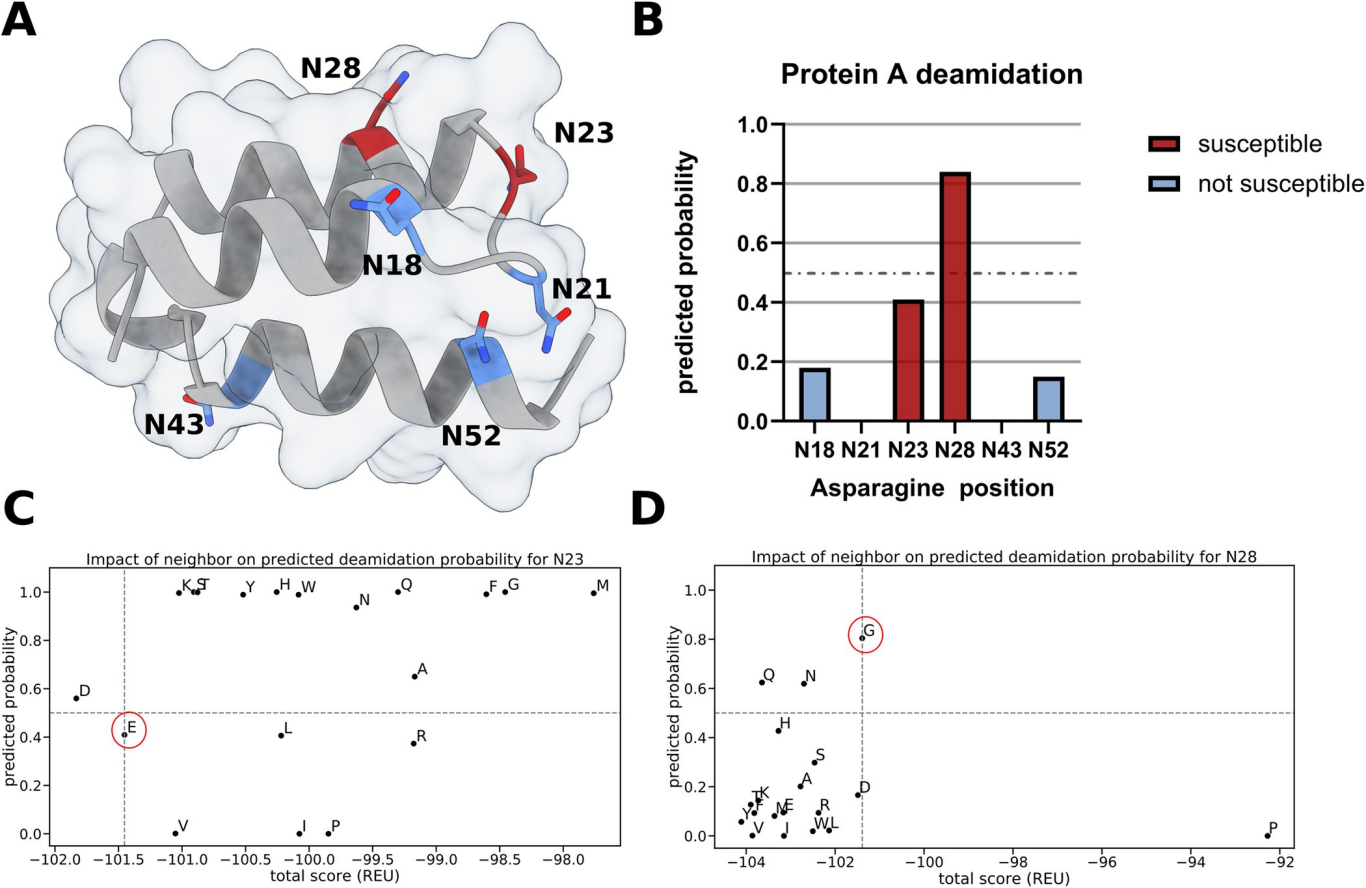
Using structure-based design to predict deamidation rates of Protein A mutations. **A).** Overview of the Protein A structure (PDB ID: 1DEE) with susceptible deamidation sites colored in red and not susceptible asparagines colored in blue. **B)** Predicted deamidation probabilities for all asparagine residues in Protein A colored by known susceptibility. The prediction threshold of 0.5 is shown as a gray dotted line. **C-D)** Predicted deamidation probabilities for mutations of residue following (n+1) the asparagine residues N23/N28 compared to the predicted stability as Rosetta energy units (where more negative equals more stable). The prediction threshold of 0.5 is shown as a gray dotted horizontal line, the vertical line identifies the total score of the native amino acid which is marked by a red circle.

As the first example we set out to predict the deamidation rate of asparagine residues in immunoglobin G binding protein A (PDB ID: 1DEE [68]). Protein A is commonly used to purify antibodies and multiple mutations were introduced to increase the stability and prevent deamidation of the residues N23 and N28 [69] (Fig 3A). We correctly predict the deamidation propensity of five out of six asparagines, with N23 showing an increased probability for deamidation but below our classification threshold (Fig 3B). The position after the asparagine residue is crucial for the deamidation probability, therefore, we used Rosetta to mutate this position to all possible amino acids except cysteine and compared their newly predicted probability to experimental data (Fig 3C and 3D). For N23 almost all n+1 mutations led to a worse Rosetta energy score and only five out of 19 led to a similar or lower predicted deamidation rate. In the case of N23 all n+1 mutations led to a decrease in predicted deamidation probability and all mutations except for proline had a better Rosetta energy score. This demonstrates that our method can be used to correctly identify deamidation sites and subsequently redesign them to sequences with reduced probabilities. A detailed capture of the protocol can be found at github.com/MeilerLab/PTMPrediction.

### Predicting glycosylation sites in Influenza hemagglutinin (HA) using structure-based analysis

**Fig 4:**
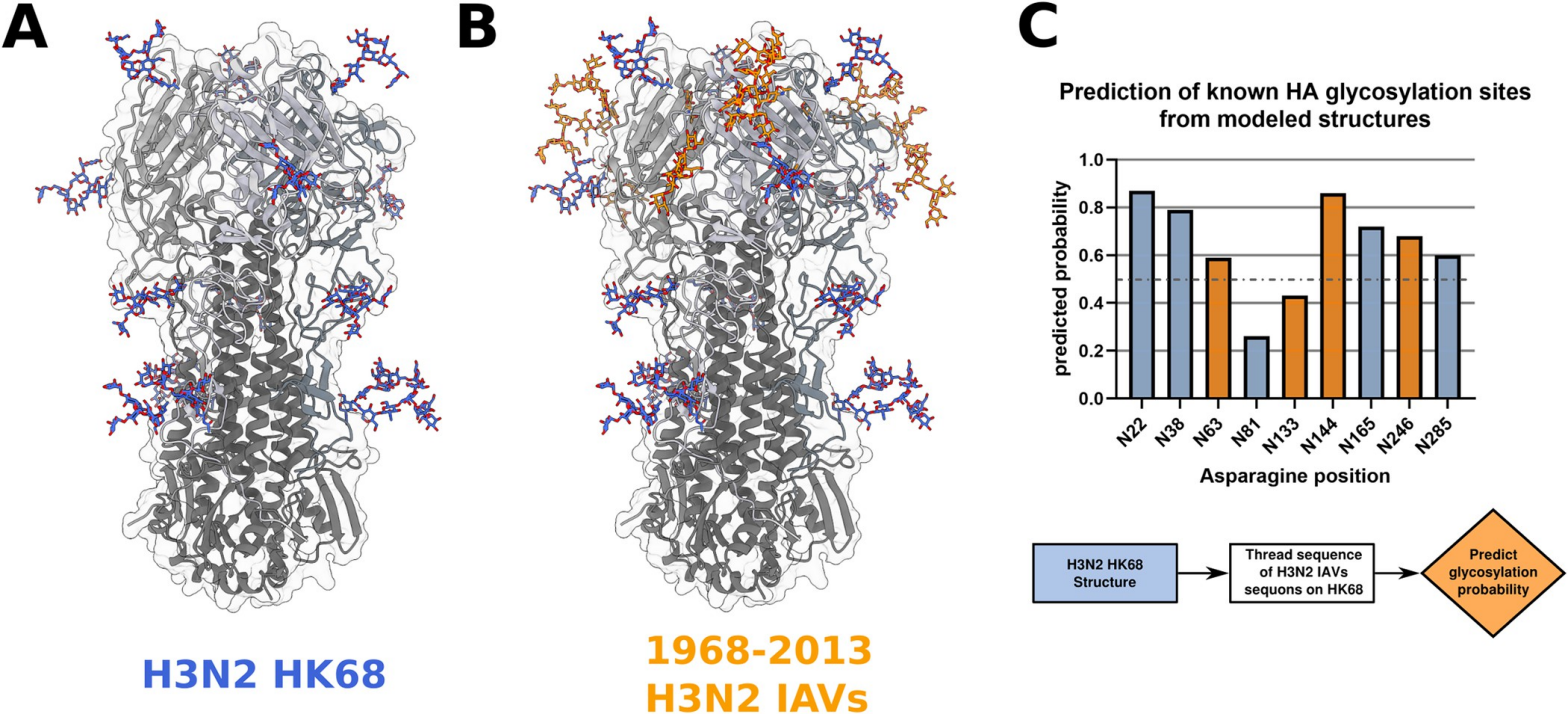
Using structure-based modeling to predict experimentally verified glycosylation sites in influenza hemagglutinin. **A).** Hemagglutinin structure of the H3N2 Hongkong 1968 (HK 68) influenza strain (PDB ID: 4FNK) with *N*-linked glycosylation sites visualized through Rosetta glycan modeling (blue). **B)** *N*-linked glycosylation sites (orange) of later observed influenza strains threaded onto the original HK 68 structure using structure-based modeling. **C**) Predicted glycosylation probabilities of known *N*-linked glycosylation sites from the early HK 68 strain (blue) or later observed strains (orange) which were modeled onto the HK 68 structure. The prediction threshold of 0.5 is shown as a gray dotted line.

As second example, we analyzed the possible occurrence of *N*-linked glycosylation sites in the H3N2 HA protein in different strains of influenza. Since the H3N2 Hong Kong 1968 (HK68) strain, Influenza gained multiple additional glycosylation sites which where experimentally validated to be occupied [70,71,72,73] (Fig 4A). Since glycans are added by the host’s cellular machinery, glycosylation facilitates viral evasion of the host immune system. For this reason, it is important to be able to predict glycosylation sites reliably to understand the function of new viral strains. We first predicted the original glycosylation sites using the structure of H3N2 HK68 (PDB ID: 4FNK [71]), correctly classifying four of the five sites. Next, we used Rosetta’s mutagenesis tools to introduce the later acquired glycosylation sequons, as well as residues two positions before or after, into the original HK68 structure and predicted their glycosylation probability. Of these four glycosylation sites, we correctly predicted three (Fig 4B). This demonstrates that we can identify *N*-linked glycosylation in a structurally heterogenous protein from modeled structures and that our method captures the evolved glycosylation sites of HA.

### Combining machine learning predictions with structure-based design to optimize the predicted phosphorylation probability of a *de novo* protein

**Fig 5:**
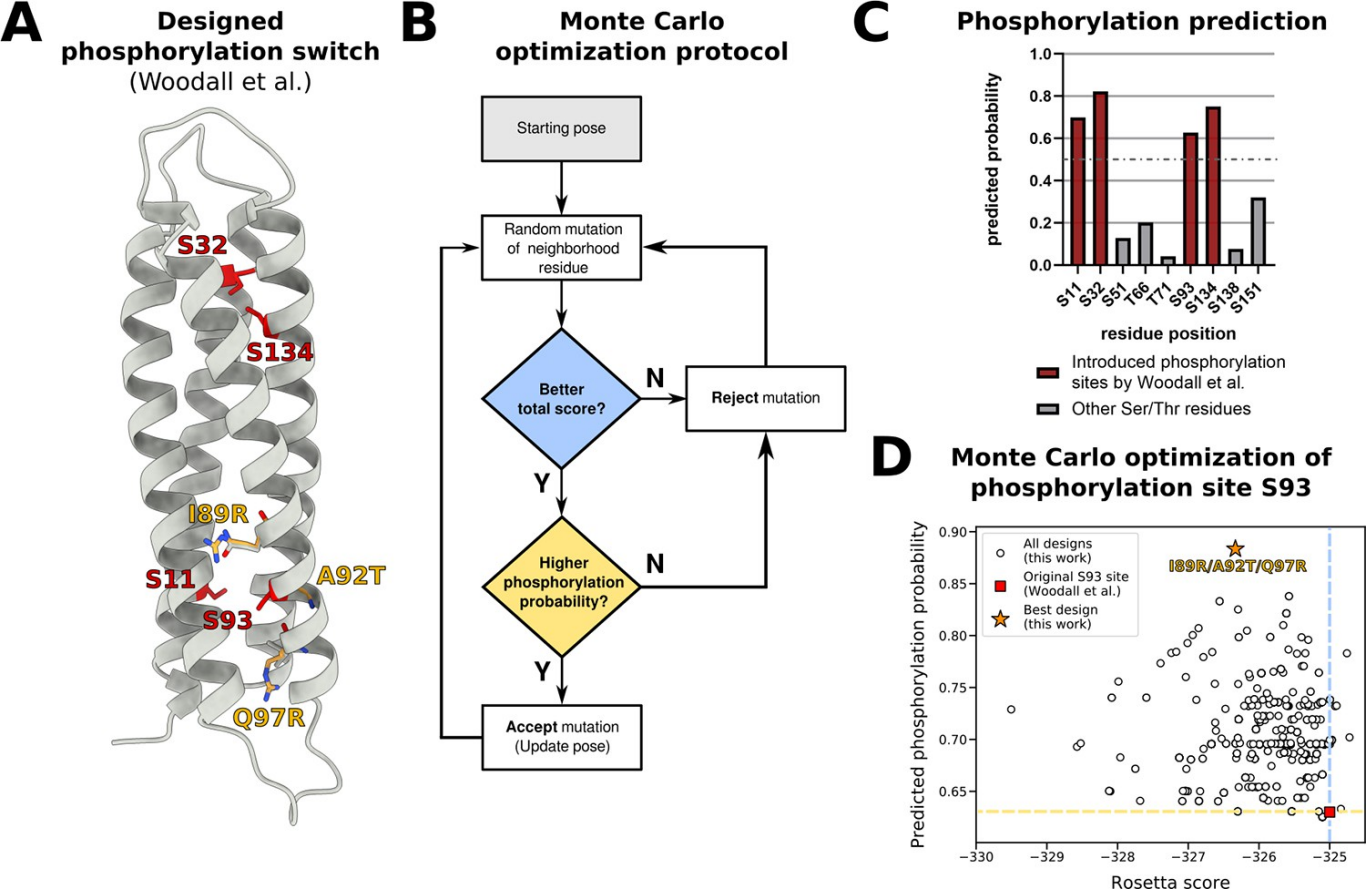
Optimizing the predicted phosphorylation probability of a *de novo* protein using structure-based design. **A).** Structure of the *de novo* serine-kinase driven protein switch from Woodall et al. [44]; originally introduced phosphorylation sites are colored red. Mutations predicted to improve the phosphorylation probability of site S93 are colored in yellow. **B)** Monte Carlo optimization protocol using the GenericMonteCarloMover, starting from the original protein structure, randomly mutating a neighborhood residue of the phosphorylation site, and then accepting or rejecting the mutation based on the Rosetta total score (using a Metropolis criterion to avoid local minima) and predicted phosphorylation probability. This inner loop is repeated 50 times and the pose with the highest phosphorylation probability is output. **C**) Predicted phosphorylation probabilities of sites introduced by Woodall et al. [44](red) and other Ser/Thr residues found in the *de novo* protein. The prediction threshold of 0.5 is shown as a gray dotted line. **D)** Results of the Monte Carlo optimization protocol for phosphorylation site S93, showing the predicted phosphorylation probability versus the Rosetta total score for 1000 trajectories. The original design is marked as red square and the best design (highest predicted phosphorylation probability) is marked as yellow star. The Rosetta score and predicted phosphorylation probability of the original design is highlighted as blue and yellow dotted line, respectively.

As a final example, we showcase how the combination with Rosetta’s structure-based design toolkit enables the optimization of Rosetta score as well as the predicted modification probability (**Fig 5**). As the PTM prediction models are implemented as SimpleMetric (in Rosetta, a module for measuring a property of a structure) they can readily be used as objective of a Monte Carlo optimization protocol. As test case, we focused on the *de novo* serine-kinase driven phosphorylation switch from Woodall et al. [44] (**Fig 5A**). To improve the predicted modification probability of a given site, we randomly mutated the neighborhood of the key residue, accepting/rejecting the mutation based on whether it improved the total score, as well as the predicted probability (**Fig 5B**). We analyzed the predicted phosphorylation of the introduced phosphorylation sites, correctly predicting them as phosphorylated and all other Ser/Thr residues as unphosphorylated, with site S93 having the lowest predicted probability of the four introduced sites (**Fig 5C**). Next, we used the Monte Carlo optimization protocol to improve the predicted probability of the phosphorylation site S93, designing a triple mutation (I89R, A92T, Q97R) which showed an improvement in predicted probability from 0.63 to 0.88 (**Fig 5D**).

## Discussion

In this work, we combined machine learning with structure-based protein design to predict and (re-)engineer PTMs in proteins. Our main result is that this combination of accurate prediction and design allows the modification of the predicted rate of PTMs occurring in proteins. We were able to predict PTM probabilities not only on native structures, but also on structures altered with Rosetta design. Interestingly, combining the prediction of certain PTMs with the prediction of other modifications increased performance for multiple cases. To do so, we pooled data for PTMs with unique modified amino acids (for example only one kind of lysine modification) and switched to a multi-class classification setting. Additionally, as this increased the number of examples for training, we added a small attention-based layer to our sequence track which is also responsible for the better performance. The improvement was especially prominent for cases with few or unbalanced data. Our initial reasoning for combining different modifications was that the surroundings of a PTM site should share a similar feature space as, e.g., a potential site must be exposed to enable enzyme binding.

In the case of Protein A deamidation, we correctly predicted the susceptibility of four out of five asparagine residues. Additionally, we could show that using Rosetta structural modelling in combination with modification prediction was able to recapitulate changes in deamidation probability. For asparagine at position 28, a mutation of its neighbor from glycine to alanine resulted in a drastically reduced deamidation probability, which is confirmed by previous experimental data [69]. In the case of influenza hemagglutinin, we were able to correctly predict four out of five glycosylation sites of the early H3N2 HK68 strain and three out of four later acquired glycosylation sites by modifying the structure of the original strain. One reason for the misclassified positions could be the inadequate modeling of the mutated backbone, which prevents accurate prediction. For the *de novo* serine-kinase driven phosphorylation switch from Woodall et al. [44] we accurately predicted the four introduced phosphorylation sites and used a Monte Carlo based optimization protocol to find mutations that increased the predicted phosphorylation probability of site S93 from 0.63 to 0.88. The best design had a Q97R mutation at the n+4 site which is in line with previous characterization of protein kinase A preferences [74]. Effective phosphorylation should increase the extent of activation in the presence of kinase and is therefore likely to improve the dynamic range of the protein switch. Taken together, these results show promise for accurate prediction not only of native, but also of designed and/or modeled proteins. As the field of PTM engineering grows more cases should become available to build a test set that goes beyond the case studies presented here. To facilitate thorough testing, a shift to also publish negative data for failed PTM engineering examples will be necessary. Additionally, we did not experimentally validate the resulting mutations of our case studies, as such a verification should test a broad set of proteins for one PTM, something that is out of scope of the current work that focuses on the prediction and engineering of many modifications. Overall, it must be pointed out that our method presented here is very challenging to benchmark, as appropriate data are not necessarily available, especially for protein design tasks. We foresee that as more data become available, our method would require updates and retraining.

Multiple other studies have worked on predicting PTMs [46,75,76,77], mainly focusing on one modification using sequence data. Here, in addition to sequence information, we leveraged the power of AlphaFold2 to enrich our features with structural data. In the case of *N*-linked glycosylation, some studies have not limited themselves to the NxT/S sequon and therefore achieve higher accuracies on their data sets [45]. Similarly, a recent study on predicting *N*-linked glycosylation used proteins that were known to be localized in the cell nucleus (and therefore never glycosylated) as negative examples [46]. While the prediction of cellular localization of proteins is interesting, this would not translate to designing new glycosylation sites. A noteworthy exception is an earlier study [78] which also used a stringent filtering approach to select positive and negative sequons based on the PDB, showing that a combination of structure and sequence features was superior to sequence features alone. Since this study was published in 2012, the number of glycosylated proteins in the Protein Data Bank has steadily increased and we showed that new progress in the field of natural language processing and the combined prediction with other PTMs further increases the prediction performance.

A limitation of our study is the quality of structures predicted with AlphaFold2. While we filtered for local and overall pLDDT, the accuracy of all predicted structural models is not guaranteed. Additionally, it has been shown that regions with low AlphaFold2 pLDDT can correlate with intrinsically disorder regions (IDRs) [79] which are known to be enriched modifications like phosphorylation or *O*-linked glycosylation [80,81]. By removing protein models with low pLDDT we might have biased our prediction for areas with well-defined secondary structure. However, the distinction between intrinsically disordered regions and low-quality regions is not possible with pLDDT alone. While this limits prediction of PTMs for IDRs it reflects the engineering use case for which the tool was created. Engineering IDRs is an exciting future prospect that will be enabled by accurate prediction of such regions.

An important caveat of this work, especially in the context of lower prediction performance for some PTMs, is the focus on PTM-aware protein engineering. We base our prediction on the local context of a potentially modified site to generalize beyond natural proteins. As the lower accuracies for some modifications highlight, our models are not intended to e.g., screen a whole proteome for glycosylation sites as models that consider homology would probably achieve higher accuracies. Instead, we focus on the downstream task of engineering particular modifications for which we optimized our prediction models, and we argue that this provides practically useful tools for protein engineering tasks. While our method allows the prediction of modifications irrelevant of protein homology or other global features like cellular localization, it therefore requires the user to be informed about the to-be engineered protein. For example, optimizing the probability of an *N*-linked glycosylation site will still not result in a glycosylated protein if the protein lacks a secretion tag or is expressed in an unsuitable system like *Escherichia coli*.

In the case of *N*-linked glycosylation, a major limitation is the availability of high-quality data. While we extensively curated our dataset, including cross-referencing UniProt data and manually checking electron densities, false negative sequons could still be present when electron densities were missing and UniProt annotations not available. One option to supplement PTMs with low data availability would have been to leverage enzyme profiling data which are available for e.g., *O*-and *N*-glycosyltransferases [82]. However, the profiling studies are based on analyzing short peptides independent from proteins, which provides information of enzyme specificity in an idealized system. Using this kind of data would therefore prevent us from using certain features calculated from protein structures, like solvent-accessible-surface-area (SASA). Additionally, taking the example of *N*-linked glycosylation, modification is far from being based on substrate recognition alone, as is shown by the preferences for loops over structured residue sites. Training models with substrate recognition data could therefore, especially in the case of PTMs with low data availability, lead to models unable to accurately predict a modification in its full protein structure context. As we think that this would be a major limitation in our downstream engineering task, we choose to limit ourselves to determined, or predicted, protein structures. To achieve better performance, data on sequons that are not occupied will be necessary, as most databases focus on positive examples.

## Conclusion

The combination of accurate prediction and structure-based design should enable the modification of existing, as well as the introduction of novel, PTMs. The potential applications of our work include, but are not limited to, glycan masking epitopes, strengthening protein-protein interactions through phosphorylation, designing PTM-dependent protein switches, as well as protecting proteins from deamidation liabilities. In conclusion, our work adds novel tools to Rosetta’s protein engineering toolbox, that allow for the rational design of PTMs.

## Methods

### Collection of proteins with PTMs

We first collected experimentally verified modifications sites from the dbPTM non-homologous benchmark dataset [61,62]. To enrich our features with structural data, we additionally used the AlphaFold2 database to download a predicted model for each protein in the dataset, filtering the models by local and overall pLDDT greater than 50. While the benchmark present in the dbPTM is already non-homologous, we clustered the sequence windows surrounding a potentially modified site (10 residues) to 90% sequence identity with CD-HIT [83] to further avoid redundancy. We calculated the SASA, dihedral angles and secondary structure for all remaining proteins using PyRosetta [63]. This procedure was done for all PTMs except for *N*-linked glycosylation and deamidation. In the case of *N*-linked glycosylation, no benchmark comparing occupied and unoccupied sequons was readily available. Therefore, we collected all eukaryotic proteins from the Protein Data Bank [64] with at least one *N*-linked glycosylation site present and searched them for additional unoccupied sequons. Next, we cross-checked potential negative sites against UniProt annotations [65,66] and removed any that were annotated as experimentally verified to be glycosylated. To further avoid false negatives, we manually checked the electron densities of all potential negatives and excluded all with ambiguous densities. As the last step we clustered the sequence identities of the sequence windows to 90% using CD-HIT. In the case of deamidation, the largest dataset available is from Delmar et al [67], however, no full sequences were published or shared on request, therefore the dataset was used without protein structure prediction or feature calculation in PyRosetta. All datasets and detailed scripts can be found at github.com/MeilerLab/PTMPrediction.

### Training of a two-track neural network to predict PTMs

We trained a two-track neural network using Tensorflow and Keras [58,84] using 10-fold cross validation through Sklearn [85] and different sampling strategies using imbalanced-learn [86]. We oversampled the positive class for all PTMs, except for phosphorylation and *O*-linked glycosylation where we under sampled the negative classes, resulting in both cases in a 1:1 ratio of negative and positive cases. Additionally, numpy [87], pandas [88,89], matplot [90] and seaborn [91] were used for data preparation and plotting. The first track of our neural network uses a sequence window of eight residues (-4/+4 around modification) as input into an embedding layer, followed by a global average pooling layer and a dense layer. The second track uses phi/psi angles of the potentially modified residue and its two neighbors, as well as the secondary structure and SASA of the potentially modified residue as input into two fully connected dense layers. The two-tracks were concatenated into one dense layer with a sigmoidal activation function outputting a probability between zero and one. In the case of training on multiple modification predictions, we added a small attention layer after the embedding layer to the sequence track, an additional fully connected dense layer to the structure track and changed the output layer to a softmax activation function (Fig 2). For the optimization we used Adam with a learning rate of 0.0001 and trained for 200 epochs with early stopping. For the multi class training we additionally used a learning rate warmup with cosine decay. A binary cross-entropy loss was applied for the single models and a sparse categorical cross-entropy loss for the multi class approach. A script to reproduce the training can be found at github.com/MeilerLab/PTMPrediction.

### Incorporation of the neural network into Rosetta

To enable rapid combination with existing design and analysis methods in Rosetta, we incorporated our prediction method as a RosettaScripts [92] element. RosettaScripts enables the rapid and flexible combination of existing protocols without proficiency in C++/Python. Therefore, we implemented feature calculation and interference in a Rosetta SimpleMetric (a module for measuring properties of a Pose) called the PTMPredictionMetric using the newly developed RosettaTensorflowManager. Full details are in **S1 Text**. Exemplary protocols to compile Rosetta with the required submodules, how to run PTM prediction and PTM design are deposited at github.com/MeilerLab/PTMPrediction.

### Deamidation rate prediction of Protein A

We collected the structure of Protein A from the Protein Data Bank (ID: 1DEE [68]) and relaxed it using FastRelax [93,94] in RosettaScripts [92]. Afterwards we predicted the deamidation probability for each asparagine using the newly developed PTMPredictionMetric which uses the described neural net. A script for this task can be found at github.com/MeilerLab/PTMPrediction. Next, we used FastDesign [95] to mutate the neighbor of N23 and N28 to all possible amino acids except cysteine and then repeated our deamidation rate prediction. ChimeraX was used to visualize the structures [96].

### Glycosylation prediction of influenza hemagglutinin

For prediction of influenza hemagglutinin *N*-linked glycosylation we first removed any ligands/glycans of the H3N2 HK68 strain (PDB ID: 4FNK [71]) and relaxed the structure using FastRelax [93,94]. We then predicted the glycosylation sites of the already present sequons using the newly developed PTMPredictionMover. Next, we introduced the sequons (including residues –2/+2) of glycosylation sites from newer strains into the original HK68 structure using Rosetta FastDesign [95], configured with a resfile specifying the particular mutations (*i*.*e*. with a fully determined sequence), and predicted their glycosylation probability. For visualization, the SimpleGlycosylateMover [54] was used to glycosylate *N*-linked glycosylation sites, and ChimeraX was used to render the resulting structures [96]. Scripts for prediction of glycosylation can be found at github.com/MeilerLab/PTMPrediction.

### Monte Carlo optimization of a *de novo* serine-kinase driven protein switch

First, we relaxed the modeled structure of pGFP-S4 from Woodall et al. [44] using FastRelax [82,83]. Next, we predicted the phosphorylation probability of all Ser/Thr residues using the newly developed PTMPredictionMover. We then created a custom RosettaScripts script incorporating the GenericMonteCarloMover to optimize the predicted probability of the phosphorylation site S93. Starting from the initial structure we randomly mutated a neighbor residue (positions 89, 92, 94, 95, 96 or 97) to any amino acid expect cysteine and then accepted or rejected the mutation based on whether it improved Rosetta total score and predicted phosphorylation probability, repeating this for 50 trials in one trajectory. Using this protocol, 1000 designs were created and ranked by improvements in total score and predicted phosphorylation probability. Scripts for the prediction and design can be found at github.com/MeilerLab/PTMPrediction.

## Supporting information

S1 Text **Table A in S1 Text.** Classes implemented for running Tensorflow models in Rosetta. **Table B in S1 Text.** Classes implemented to support the PTMPredictionMetric. **Table C in S1 Text.** Summary of positive and negative examples for each PTM type
(DOCX)
