# Supplementary material for: Combining machine learning with structure-based protein design to predict and engineer post-translational modifications of proteins
Source: PLoS Comput Biol. 2024 Mar 14;20(3):e1011939. doi: 10.1371/journal.pcbi.1011939 (PMC10965067; doi:10.1371/journal.pcbi.1011939)
Supplement: S1 Text — Table A in S1 Text. Classes implemented for running Tensorflow models in Rosetta. Table B in S1 Text. Classes implemented to support the PTMPredictionMetric. Table C in S1 Text. Summary of positive and negative examples for each PTM type (DOCX) [file pcbi.1011939.s001.docx]

**Supplementary Information**

**Implementation of the RosettaTensorflowManager**

The ability to combine modern machine learning (ML) methods with traditional protein modelling tools is essential to advance protein modelling [57]. In the case of this work, deep neural networks able to predict protein posttranslational modification (PTM) sites and propensities proved to be an important complement to existing protein design algorithms and protocols, permitting PTM-aware design. ML models typically represent data very differently than conventionally-written computer programs, however, necessitating a programming framework that maximizes cross-compatibility while minimizing developer error. Tensorflow’s C language API allows ML models created with that package to be run in the context of a conventional program, but lack many of the features of higher-level languages, such as C++, that ensure automatic memory management and prevent common developer mistakes leading to bugs or cryptic failures. Additionally, these methods provide no built-in support for efficiently loading models from disk (without repeated reads) in the context of a long traditionally-written program that might make multiple calls to a model, or call multiple models over the course of its execution. For this reason, we wrapped calls to the Tensorflow API in a set of C++ classes that centralize the direct API calls while providing a safer external API for Rosetta developers.

The classes implemented for running Tensorflow models in Rosetta are shown in **Table A**. All of these classes are located in the basic::tensorflow_manager namespace, and are located in Rosetta/main/source/src/basic/tensorflow_manager. Additional utility functions are implemented in util.cc and prototyped in util.hh, in the same namespace and directory.

| **Table A:** Classes implemented for running Tensorflow models in Rosetta.**Class Name** | **Purpose** |
| --- | --- |
| RosettaTensorflowManager | A global manager that fields requests for particular Tensorflow ML models, in the form of Tensorflow sessions. Ensures that models are loaded from disk once, lazily, and in a threadsafe manner, and are cached after initial load to fill subsequent requests, minimizing slow disk access in intensive Rosetta protocols on shared filesystems on large computing clusters. |
| RosettaTensorflowProtocolBase | A pure virtual base class for Rosetta protocols that use Tensorflow. These protocols define the methods for converting Rosetta objects into Tensorflow input tensors and for converting Tensorflow output tensors into Rosetta objects. |
| RosettaTensorflowSessionContainer | A container for a Tensorflow model or session. This object uses the “resource acquisition is initialization” (RAII) C++ paradigm, loading the session in a threadsafe manner on container initialization and safely performing cleanup on container destruction. Session containers are not created directly by Rosetta developers, but are requested from the RosettaTensorflowManager by filename or other key. The session container provides a run_session() method that accepts an input RosettaTensorflowTensorContainer and produces an output RosettaTensorflowTensorContainer. An additional multirun_session() method operates on a vector of inputs to produce a vector of outputs. Member functions also exist to run models that take multiple input tensors or produce multiple output tensors. |
| template <typename T>  RosettaTensorflowTensorContainer | A RAII container for Tensorflow tensors that serve as both input and output into Tensorflow sessions or models. The container ensures safe initialization and cleanup of the memory needed for the tensor, and provides convenience methods for accessing entries in the tensor using 1-based Rosetta indexing instead of 0-based Tensorflow indexing. This is a template class to permit different datatypes (integers, floating-point numbers, double-precision floating-point numbers, *etc*.) to be passed to models. Rosetta protocols that use Tensorflow models must instantiate RosettaTensorflowTensorContainers and convert Rosetta inputs into input tensors, and must also convert output tensors encapsulated in RosettaTensorflowTensorContainers back into appropriate Rosetta objects. |
| template <typename T> TFDataTypeDetector | A class for managing allowed datatypes compatible with Tensorflow. This is used internally within the RosettaTensorflowTensorContainer class, and is generally not a class with which developers need to interact directly. |

**Implementation of the PTMPredictionMetric**

In Rosetta, SimpleMetrics are modules which measure properties of a structure (or Pose) and return strings, values, or other data reflecting the measurement. We implemented a SimpleMetric called the PTMPredictionMetric which takes as input a protein structure or Pose and produces as output a score, ranging from 0 to 1, for each residue based on the likelihood that that position bears a user-specified type of PTM. Currently supported posttranslational modifications include: acetylation, argenine methylation, citrullination, crotonylation, asparagine deamidation, glutamate γ-carboxylation, glutarylation, glutathionylation, hydroxylation, lysine methylation, malonylation, N- and O-linked glycosylation, phosphorylation, S-nitrosylation, succinylation, sumolation, and ubiquitionation. Internally, the PTMPredictionMetric runs a Tensorflow deep neural network to make its prediction; however, the user need not interact with this neural network directly or have any understanding of deep learning in order to use the metric. Since the internal architecture supports multiple Tensorflow models, additional PTMs can be added in the future.

All classes for the PTMPredictionMetric are implemented in the protocols::ptm_prediction namespace, and are found in C++ source files in Rosetta/main/source/src/protocols/ptm_prediction. The implemented classes are shown in **Table B**.

**Table B:** Classes implemented to support the PTMPredictionMetric

| **Class Name** | **Purpose** |
| --- | --- |
| PTMPredictionMetric | The metric, derived from the Rosetta SimpleMetric base class, which computes per-residue PTM likelihoods given an input Pose. This class provides an XML, Python, and C++ user interface for setting options, such as which PTM type is being predicted and what residue selector will be used to select a subset of residues for the prediction. Internally, it stores a shared pointer to a PTMPredictionTensorflowProtocolBase object, permitting any derived class to be stored and permitting future extensibility (*i.e.* allowing future versions of the protocol class to replace the current version). This SimpleMetric provides an apply() function that takes as input a Pose and provides as output a map of residue index to a double-precision floating-point value representing the probability of observing the given PTM at a particular residue. |
| dPTMPredictionTensorflowProtocolBase | A common, pure virtual base class for PTM-predicting protocols. Derived classes must implement a compute_ptm_probability() function that takes as input a Pose, a residue selector, and a PTM type, returning a map of residue index to PTM likelihood score. The pure virtual class permits later implementation of additional protocols, trained on newer training data and/or supporting an expanded range of PTMs. Internally, this stores a RosettaTensorflowSessionContainer containing the trained PTM prediction model, retrieved from the RosettaTensorflowManager. |
| PTMPredictionTensorflowProtocol | The current implementation of the PTM-predicting protocol, providing methods for converting a Pose into suitable tensor representations for input into the deep neural net, and for converting the tensor output into a map of amino acid residue index to PTM likelihood. Internally, this calls run functions in the RosettaTensorflowManager to run the multi-input model on multiple input tensors, producing output tensors. |

Documentation for the PTMPredictionMetric is available at https://new.rosettacommons.org/docs/wiki/scripting_documentation/RosettaScripts/SimpleMetrics/simple_metric_pages/PTMPRedictionMetric Information on the RosettaScripts XML interface may also be obtained by running the RosettaScripts application with the flag -info PTMPredictionMetric.

**Compiling Rosetta with Tensorflow support**

The default compilation of Rosetta has no support for Tensorflow ML models. To compile Rosetta with Tensorflow support, one must follow the steps listed below to link against the Tensorflow API libraries. These instructions are printed automatically in an error message if one tries to use Rosetta modules dependent on Tensorflow ML models from a build of Rosetta that does not include Tensorflow support:

1. The precompiled Tensorflow 1.15 libraries must be downloaded and unzipped into a suitable directory. This directory must be added to the $LIBRARY_PATH and $LD_LIBRARY_PATH environment variables on Linux or Windows, or to the $LIBRARY_PATH and $DYLD_LIBRARY_PATH environment variable on Macintosh. At the time of this writing, Tensorflow 1.15 libraries are available at the following locations:

**Linux/CPU**: https://storage.googleapis.com/tensorflow/libtensorflow/libtensorflow-cpu-linux-x86_64-1.15.0.tar.gz

**Linux/GPU**: https://storage.googleapis.com/tensorflow/libtensorflow/libtensorflow-gpu-linux-x86_64-1.15.0.tar.gz

**Windows/CPU**: https://storage.googleapis.com/tensorflow/libtensorflow/libtensorflow-cpu-windows-x86_64-1.15.0.zip

**Windows/GPU**: https://storage.googleapis.com/tensorflow/libtensorflow/libtensorflow-gpu-windows-x86_64-1.15.0.zip

**MacOS/CPU**: https://storage.googleapis.com/tensorflow/libtensorflow/libtensorflow-cpu-darwin-x86_64-1.15.0.tar.gz

**MacOS/GPU**: None available.

2. The file Rosetta/main/source/tools/build/user.settings must be edited. The following lines must be uncommented (*i.e.*, the octothorpe at the start of the line removed):

import os

‘program_path’ : os.environ[‘Path’].split(‘:’),

‘ENV’ : os.environ,

3. Rosetta must be compiled with extras=tensorflow or extras=tensorflow_gpu appended to the scons command for CPU- and GPU-supporting versions of Tensorflow, respectively. For example, ./scons.py -j 8 mode=release extras=tensorflow bin.

4. Rosetta executables with Tensorflow support will appear in the Rosetta/main/source/bin directory. The name of each application will have the pattern <application_name>.tensorflow/tensorflow_gpu.<OS><compiler><mode>. For instance, the typical release-mode CPU compilation of Tensorflow on Linux of the RosettaScripts application would be rosetta_scripts.tensorflow.linuxgccrelease.

The above steps can be combined with other “extras” specified at compile time, such as support for multi-process execution with the Message Passing Interface, or MPI (extras=mpi,serialization,tensorflow) or support for multi-threading (extras=cxx11thread,tensorflow).

**Table C:** Summary of positive and negative examples for each PTM type

| PTM | positive samples | negative samples | total samples |
| --- | --- | --- | --- |
| Hydroxylation | 393 | 744 | 1137 |
| Gamma-carboxyglutamic-acid | 157 | 160 | 317 |
| Lysine Methylation | 5522 | 9582 | 15104 |
| Malonylation | 6289 | 11295 | 17584 |
| Arginine Methylation | 3065 | 10911 | 13976 |
| Crotonylation | 23 | 122 | 145 |
| Ubiquitination | 5174 | 3767 | 8941 |
| Succinylation | 3657 | 3690 | 7347 |
| Glutathionylation | 3068 | 4710 | 7778 |
| Sumoylation | 1806 | 8246 | 10052 |
| *S*-nitrosylation | 2327 | 3426 | 5753 |
| Acetylation | 11177 | 3279 | 14456 |
| *O*-linked Glycosylation | 1048 | 14878 | 15926 |
| Phosphorylation | 2572 | 58768 | 61340 |
| Glutarylation | 1041 | 1879 | 2920 |
| Citrullination | 59 | 881 | 940 |
| *N*-linked Glycosylation | 2115 | 355 | 2470 |
